# Value of First Aid in the Management of Mental Cases and Hospitals.—Part II
*Abstracts of a paper read in this Section by Sir Robert Armstrong-Jones, M.D., Temporary Lieut.-Colonel R.A.M.C., at the College of Ambulance, Vere Street, London, W., Sir James Crichton Browne, M.D., F.R.S., in the chair.


**Published:** 1918-07-27

**Authors:** 


### Value of First Aid in the Management of Mental Cases and Hospitals.*—PART II.

The work of efficient nursing has made remarkable progress during the last quarter of a century, and in no class of hospital has this been more marked than in our large mental hospitals. I humbly claim, with my medical colleagues, to have helped in this, for the Claybury Mental Hospital in 1893 was the first public asylum in the County of London to adopt the teaching and to apply training to its nurses upon an approved syllabus, which included in its scheme first-aid work, home nursing, and hygiene. I am able to add that this training was warmly approved by the L.C.C., who were the administrative authorities responsible for the care of poor mental patients in London, who number to-day no less than 20,000 persons. To-day there is not a single mental hospital in the United Kingdom which does not apply a systematic course of instruction and training to its nursing staff, and there are, since the first examination by the Medico-Psychological Association in 1891, over 12,000 trained mental nurses who have received certificates of proficiency based primarily upon the principles of first aid, domestic hygiene, and sick nursing.

It is claimed for those who have received instruction that they are taught to be more observant, to note the signs of injury and disease, and to recognise the cause; for to know the cause is to make prevention more easy. Also they are more tactful in their dealings with their patients, an inestimable quality in the mental nurse; they become more thoughtful, and they impart greater confidence to those among them. It is impossible to overstate the value of intelligent lay help, and one reason why first-aid work has become so widely appreciated is the fact that there is a uniform system; there is no over-centralisation; every mental hospital can carry out its own scheme of instruction provided it is in accord with a common syllabus for which there is provided an inexpensive practical handbook. The great aim of the teaching is to assist Nature's method of restoration and repair, to ease pain, to prevent further damage and mischief, and to ascertain which of several injuries presses most for treatment, and to apply this with skill.

I hope I may be permitted to quote a few instances from my personal experience at Claybury to show the inestimable value of sound training in first principles and based upon the first-aid teaching carried out at this College. A party of mental patients went out shopping one afternoon in the care of three nurses, when one of them suddenly jumped into the deepest part of the village pond. She was rescued in a state of unconsciousness, but the nurses at once performed artificial respiration and brought the patient round. On another occasion, when the nurse was measuring out medicines in the store-room of a ward, a patient rushed in and seized a bottle of paraldehyde—a sedative prescribed for sleeplessness and excitement. She drank a large quantity, but the nurse promptly administered an emetic and kept the patient alive until the arrival of the doctor. Whilst at dinner, a patient in one of the wards suddenly seized a piece of meat from another's plate and endeavoured to swallow it whole, but it stuck in the pharynx and she was unable to remove it; symptoms of asphyxia set in, the patient became black in the face and ceased breathing, but the nurse in attendance, with great presence of mind and some difficulty, removed the obstruction, laid the patient down, performed artificial respiration, and thus saved her life. A patrolling night nurse slipped a knife she was using for cutting bread into her pocket and went to the "pegging" clock near the bedside of a patient, who, unseen and unfelt by the nurse, drew the knife from her pocket and concealed it in her bed; later she cut her throat severely with it, but she was almost immediately afterwards seen by the nurse in a veritable pool of blood, much exhausted and still bleeding. The nurse drew the wound together, made pressure on the bleeding point, arrested the hæmorrhage, and saved the patient's life. One afternoon, whilst the patients and nurses were out in the gardens, a carter on his way to the farm, and sitting on the shaft, was seen suddenly to fall and the wheel of the cart went over him. Although the farm road was over a quarter of a mile away, the nurses took a stretcher and some rough splints to the spot and found he had sustained a serious injury to both legs. He was "put up" for broken thighs and carried into the wards, where his injuries were treated. Had he fallen into untrained hands he would probably have sustained a compound fracture of the femur, and he would not have enjoyed the recovery which trained assistance enabled him to secure. A man, seventy-eight years of age, attempted to commit suicide by placing his head in the kitchen sink full of water. He was apparently dead when discovered, but artificial respiration was kept up by relays of men who had been instructed in the application of this treatment, and the patient was brought round. A patient attempted suicide by gas-poisoning. He deliberately made a mask for himself out of some flannelette material. He attached one end of a rubber tube to the gas-tap, but passed the free end through a hole in the mask and turned on the gas. He was found apparently dead, but artificial respiration restored him, and he was discharged six years afterwards. Another patient attempted suicide by cutting the arteries in his legs with a sharp piece of steel. He was discovered bleeding freely and lying unconscious in a wood. The hæmorrhage was arrested and the patient was restored by first-aid treatment. He was discharged recovered some time afterwards. A house painter slipped off a ladder and fell a distance of 40 feet. He was terribly injured: both arms, both legs, and his ribs were broken. He was most carefully tended by first-aid help, supported and carried on an ambulance stretcher to a bed on the ground floor, and his injuries tended to by a trained staff until the doctor arrived. He made a good recovery, and, although somewhat crippled and deformed, he now bicycles daily to and from his work. Whilst motoring to North Wales last summer I was suddenly requested to help to restore a drowning man in the river at Bettws-y-coed, but when I arrived there I found one of my old trained mental nurses performing artificial respiration, which we kept up until the man was restored. These are all instances of unusual and unexpected emergencies when life was saved. A knowledge of the mechanism of the circulation and of respiration and digestion, a simple acquaintance with the bony skeleton, help to the ministration of reasonable practical relief in the direst needs, but this instruction contemplated by the St. John Ambulance Association and the Red Cross Society, which is daily carried on at the College, aims at even more than first-aid work. It teaches the laws of health, household economy, domestic cleanliness, all based on science; sick cookery, and the elementary care of infectious fevers, including the avoidance of their complications and the application of simple methods of disinfection. I maintain that these should not only be taught to the staff, but that such a mental discipline should be applied to all convalescing patients, and I think such training would not only tend to expedite convalescence whilst under treatment, but also to prevent relapse upon discharge; besides, such a training would be the best health missionary work that could be instilled into the poorer homes, and by taking on this work the mental hospitals of this country would come to be regarded not only as curative establishments, but also as educational centres whose influence would extend to the home and would reflect credit upon our teaching and nursing staff. We wait eagerly for a more direct recognition of the work done by nurses in our mental hospitals. The Order of the Royal Red Cross, instituted by Queen Victoria in 1883, has been conferred upon ladies who have devoted themselves to special work of relief in pre-war days; but many brave deeds and noble acts of devotion and self-sacrifice have been and are daily performed by the nurses in our mental hospitals, where special recognition is almost unknown. I feel convinced, if our mental hospitals would become schools for the re-education of our patients as well as for the training of the staff, on the lines carried out in this College, to use the words of Parkes, that life would be rendered fuller and happier, health would be more perfect, and death would become more remote.

* Abstracts of a paper read in this Section by Sir Robert Armstrong-Jones, M.D., Temporary Lieut.-Colonel R.A.M.C., at the College of Ambulance, Vere Street, London, W., Sir James Crichton Browne, M.D., F.R.S., in the chair.

